# The Treatment of Acute Intussusception

**Published:** 1908-06-20

**Authors:** H. F. Parker

**Affiliations:** Hon. Medical Officer to the Royal Surrey County Hospital, Guildford.


### SPECIAL ARTICLE.

### THE TREATMENT OF ACUTE INTUSSUSCEPTION.

By H. F. Parker, M.D., B.C. (Cantab.), M.R.C.S. (Eng.), Hon. Medical Officer to the Royal Surrey County Hospital, Guildford.

In discussing the treatment of acute intussusception we may begin by considering what is the ordinary course of the disease in untreated cases. In the first place spontaneous reduction, if it occurs at all in correctly diagnosed cases, is so rare that its further consideration need not detain us. Secondly, a natural cure may result by means of the separation of a gangrenous intussusception, whilst at the same time continuity of the gut is secured by adhesions at the neck of the intussusception between the peritoneum of the entering layer and that of the reflected layer of the sheath. Treves* states that "elimination of a gangrenous intussusception takes place in about 42 per cent. of (irreducible) cases, and the death rate among those in whom it does occur is over 40 per cent." This process of spontaneous cure, however, seldom takes place in infants, and in any case an annular constriction of the intestine is liable to form and cause symptoms of obstruction at a later date. In the great majority of cases death occurs within three or four days, and it is not right, therefore, to leave a case to nature unless the condition of the patient absolutely forbids surgical interference.

Before the present era of abdominal surgery the usual treatment of acute intussusception was attempted reduction by distension of the large bowel with air or water, introduced per anum, with simultaneous manipulation of the abdomen. In early cases this method of treatment was not infrequently successful. It has, however, deservedly fallen into disrepute as a routine measure for the following reasons:

(1). The percentage of recoveries is small even in cases so treated in quite an early stage. Mr. Bernard Pitts gives the following statistics of cases admitted to St. Thomas's Hospital.† Previous to the year 1898, of 105 children under the age of twelve treated for acute intussusception, thirty-six recovered, thirteen of these after treatment by inflation and manipulation, and twenty-three after laparotomy. Since that date and up to the year 1901, out of forty-nine cases one only was treated, and that successfully, by the injection of water; of the remaining forty-eight on whom primary laparotomy was performed twenty-seven died and twenty-one recovered. As far as these figures go, therefore, they point to a considerably greater saving of life among cases treated by immediate abdominal section as compared with those treated by inflation.

(2) Injections are useless in certain varieties of intussusception, namely, in the enteric and ileo-colic forms, comprising as they do respectively about 30 per cent., and 8 per cent. of all cases.‡ Moreover, the diagnosis of the particular variety present is not always possible before operation; occasionally, also, a double intussusception is present; or, again, the disease may be caused by the dragging of a polypus, or be associated with the presence of a Meckel's diverticulum, which conditions would be likely to cause recurrence of the trouble.

(3) Even after apparent reduction by this method it is very common for the symptoms to recur; and this is a very serious objection to the treatment. The fact is that the last inch or so of the intussusception is by far the most difficult part to reduce on account of the œdematous condition of its apex, the presence of peritoneal adhesions, and the damaged condition of the tissues which renders unjustifiable the use of any considerable degree of pressure by water or air. One cannot, therefore, be certain whether reduction is complete or not for perhaps some hours; and if, in consequence of recurrence, an operation is subsequently called for, the additional shock produced may prove fatal.

Nevertheless, in spite of these objections, there is a limited field of usefulness open to the method of injection:

(1) In mild cases it should be tried where the friends of the patient refuse to allow any surgical operation, or where, *e.g.* in the absence of assistance, an operation is impossible.

(2) It may be tried alone in very early cases, *i.e.* those of less than twelve hours' duration, provided that the symptoms are not severe.

(3) If in a fairly early case the intussusception appears to be travelling along the transverse or the descending colon, a preliminary injection may be tried in order to restrict the area of subsequent operation, and to enable the surgeon to make the incision over the tumour instead of in the middle line. No time, however, should be wasted in attempting to do this unless there is a fair prospect of success.

(4) Injection may be carried out by an assistant during the progress of an abdominal section, the surgeon himself manipulating the tumour with his fingers inside the abdominal cavity. This "combined method," as it is called, is very useful in suitable cases and, if carried out with care, is accompanied by comparatively little shock. It is only suitable, however, in fairly early cases, and is only applicable to the ileo-cæcal and colic forms.

In the great majority of cases the proper treatment is immediate abdominal section, followed by reduction of the gut if possible; or, should it be found to be irreducible, by one or other of the operations to be presently described. Whatever mode of treatment seems indicated in any particular case, the general consensus of opinion now is entirely in favour of all cases being placed under the care of a surgeon from the onset. Before proceeding to give details of operative treatment, a few general remarks may be made.

In cases of doubtful diagnosis an anæsthetic may materially assist the surgeon in coming to a conclusion by revealing a latent tumour in the abdomen. A careful rectal and bimanual examination should be made. Even under an anæsthetic a tumour may fail to be detected should it be deeply situated beneath the liver.

### Measures for the Prevention of Shock.

The shock to an infant suffering from acute intussusception is very great, and therefore a long operation is generally impossible. The degree of shock depends upon the age and physique of the child, and also upon the severity of the lesion. Cases of ileo-colic intussusception are accompanied by greater shock than the other varieties owing to the more complete strangulation of the intussusception by the ileo-cæcal valve. As a general rule, shock is proportional to the duration of the symptoms; but this is not an absolute rule. To reduce shock to a minimum the following measures should be taken:

(*a*) The patient should be placed on a warmed table or hot-water cushion in a well-warmed room. The limbs should be wrapped in cotton-wool, and the surface of the body should be exposed as little as possible.

(*b*) The surgeon should endeavour to use the utmost speed that is consistent with due care in dealing with the damaged tissues. He should aim, if possible, at completing the operation within twenty minutes. Any prolonged operation, in fact, in an infant is so generally followed by death from shock that the importance of this cannot be exaggerated. Here we may mention the necessity of having a skilled and reliable assistant.

(*c*) The personal equation of the anæsthetist is of considerable moment. The administration should not be commenced until the surgeon is quite ready, and the anæsthesia should be as light as possible. In regard to the anæsthetic employed we have personally a strong preference for ethyl chloride if the administrator is thoroughly versed in its use and is prepared to give it continuously by an open method, using, *e.g.*, a Brewer's mask. The shock resulting from its use is much less than that from chloroform; its effect on the heart and on respiration is almost nil, and sufficient muscular relaxation can be maintained in an infant with a very small quantity. Recovery from its effects is rapid, and subsequent vomiting is either trifling or absent. We may now proceed to consider in detail the different modes of treatment.

### Distension of the Colon by Air or Liquid.

Probably a hot nutritive fluid, such as hot milk, is better than either air or water; and a liquid is perhaps easier to deal with than air. The patient being anæsthetised and the pelvis well raised on a cushion the fluid should be carefully and slowly injected into the rectum by means of a funnel and soft rubber tube, the latter being passed for a distance of several inches into the bowel, and the former being held not more than two feet above the level of the body. Excessive pressure has occasionally in careless hands caused rupture of the intestine. If at hand, a Lund's Insufflator may be used in order to prevent the escape of fluid per anum; but it should be provided with a tube and funnel and not with the Higginson syringe that is usually depicted in books. Some fifteen to twenty minutes should be spent upon the proceeding, and at the same time any abdominal tumour should be kneaded with the fingers, the progress of reduction being carefully observed.

Even if reduction be apparently complete, it will usually be advisable to make an incision $1\frac{1}{2}$ in. long in the region of the cæcum, which should then be brought into view with the finger in order to ascertain that reduction has been achieved. Should injection alone fail, or should the symptoms recur after apparent success by this method, no time should be lost in performing laparotomy.

### Laparotomy.

If a localised tumour be present on the right side of the abdomen it is best to make the skin incision in the right semilunar line. If, on the contrary, the outline of the tumour be indefinite, or if a gangrenous condition of the gut be suspected, it is better to make it close to the middle line through the sheath of the right rectus muscle and just below the umbilicus. The incision at first should be short —1½ to 2 in.—but there should be no hesitation in prolonging it if necessary.

The fibres of the abdominal muscle should be separated rather than divided in order to minimise the risk of prolapse of the intestines should suppuration occur. The sides of the wound must be well retracted, and the intestines packed out of the way by abdominal sponges or pads, as their prolapse outside the abdomen is highly undesirable. If there be much distension of the gut it is sometimes advisable to evacuate some of the contents through a minute incision, which is then closed by a Lembert's suture.

The condition of the neck of the tumour should be examined in the first place. If it shows signs of gangrene no attempt at reduction must be made. Otherwise the fingers should feel for the apex of the intussusception, and should then proceed to compress it gently and gradually within its sheath, so as to undo the process of intussusception.

Traction on the entering layer is to be condemned as both useless and dangerous on account of the danger of splitting the peritoneal and even the other coats of the sheath at the neck of the tumour. Should this accident happen a few Lembert's sutures should be inserted.

The most difficult part of the operation is the reduction of the last inch or so of the intussusception, though this is usually possible in cases that have not lasted for more than forty-eight hours.

In infants under two years of age excision of the gut for irreducible intussusception is so uniformly fatal that it would seem justifiable to use a certain amount of force in attempting reduction.§

Should the tumour be not wholly reducible and yet not gangrenous the best treatment (except in the case of infants) is to resect the irreducible portion *in situ*. This has been done successfully by Mr. Barker in some cases by drawing the intussusception through the anal aperture and then cutting off the lower portion.|| In general, however, the following operation is to be recommended¶:

The intussusception having been reduced as far as possible, the entering and ensheathing layers are sutured together at the neck of the tumour by a fine continuous suture. Clamps are then applied to the intestine above and below the intussusception. Next, the sheath is incised longitudinally below the neck on the side opposite to the mesenteric attachment, and the intussusception is drawn down through this incision. Every care is taken to avoid fouling the peritoneum, and this is much facilitated by the use of clamps. The proximal end of the intussusception is then united by a few catgut sutures to the reflected layer of the sheath, after which the distal end or apex is cut off below the sutures. The incision in the sheath is then closed in the usual manner.

In cases where the gut is gangrenous the prospect of recovery is practically hopeless in infants under two years of age, and it is seldom that even older children recover. For this condition there is a choice of operations, the selection depending usually upon the state of the patient.

1. Excision of the damaged bowel, the cut ends being united either by Maunsell's method or by simple end-to-end anastomosis. In this latter case two rows of continuous sutures should be used, the inner one including the whole thickness of the wall of the bowel, and the outer one penetrating the peritoneal and muscular coats only. This operation is much more severe than the following; but, if successful, it gives the more satisfactory result.

2. A more rapid, and therefore less severe, operation is excision of the damaged bowel, followed by the insertion of Paul's tubes. These should be brought outside the abdomen, preferably through a separate small incision in such a situation that there is as little tension as possible on the sutures that unite the ends of the bowel to the parietal peritoneum. A subsequent operation is needed to close the artificial anus thus made. This method, however, cannot replace that of immediate suture in cases of the enteric form of intussusception, unless this occurs quite low down in the small intestine.

3. The formation of an artificial anus at some distance above the intussusception. The danger of perforation at the neck of the tumour should be minimised by slightly increasing the extent of the invagination and uniting the two peritoneal surfaces at the neck by a few catgut sutures. The idea of this method is to relieve obstruction by the formation of an artificial anus in the hope that the gangrenous intussusception may slough off and be passed per rectum. A permanent fæcal fistula, however, is liable to result from this proceeding.

4. Since a few cases left to nature are cured by spontaneous separation of the intussusception, it may be advisable to spare the patient the shock of any further operation beyond that of inserting a few sutures at the neck of the tumour, as in the preceding operation, and closing the wound.

The suturing of the abdominal wall should be done as carefully as time allows. A serious late complication is the bursting open of the wound with prolapse of the intestine in consequence of suppuration. It is advisable, therefore, when possible, to use buried sutures, and also to leave the superficial sutures until the wound is soundly healed.

Measures should be taken to guard against death from shock, *e.g.* by enemata of hot peptonised milk and brandy, transfusion with normal saline solution, strychnine administered hypodermically, etc.

It is important to administer opium or morphia in small and appropriate doses in order to lessen peristalsis, and it may be advisable to perform the first dressing with the patient under the influence of morphia and of an anæsthetic.

Careful attention should be paid to diet, and all causes of gastro-intestinal disturbance should be avoided. The bowels should be kept acting regularly, but only the mildest aperients should be given.

* Treves' "System of Surgery" (1896), vol. ii., p. 653.

† "British Medical Journal," September 7, 1901, p. 574.

‡ Treves' "System of Surgery" (1896), vol. ii., p. 635.

§ "Lancet," December 17, 1904, p. 1719.

|| "British Medical Journal," 1892, vol. ii., p. 1226.

¶ Cheyne and Burghard's "Manual of Surgical Treatment," part 6.

